# Loneliness in older women: a multidimensional population-based study of social, health, and life-course factors

**DOI:** 10.3389/fragi.2026.1858726

**Published:** 2026-07-29

**Authors:** Esther Cabrera, Meritxell Puyané, Marc Terradellas, Esther Limón, Ester Mateo, Enric Camón, Carme Planas, Rosario Delgado, Chaimae Lamrani, Carolina Chabrera

**Affiliations:** 1 Research Group on Chronic Care and Health Innovation (GRACIS), TecnoCampus, Universitat Pompeu Fabra, Barcelona, Spain; 2 Department of Social Welfare, Mataró City Council, Barcelona, Spain

**Keywords:** community-dwelling, depressive symptoms, healthy ageing, loneliness, older women, social isolation, Spain

## Abstract

**Introduction:**

Loneliness in older women is a major public health concern in ageing societies and a key determinant of healthy ageing. Despite its multidimensional nature, evidence on the relative contribution of social, health, and individual factors remains limited, particularly from a gender perspective.

**Methods:**

A cross-sectional population-based study was conducted in Catalonia using random sampling from the municipal census. Data were collected through face-to-face home interviews with 511 women. Loneliness was assessed with the 10-item Spanish version of the UCLA Loneliness Scale and analyzed as a binary outcome (moderate/severe vs. low or no loneliness). Explanatory variables were grouped into sociodemographic, social, clinical, functional, and quality-of-life domains. Crude and adjusted prevalence ratios were estimated using Poisson regression with robust variance.

**Results:**

Overall, 18.6% of participants reported moderate or severe loneliness. In crude analyses, loneliness was more frequent among women living alone, with lower income, weaker community belonging, greater social vulnerability, depressive symptoms, frailty, poorer physical performance, higher fall risk, and lower quality-of-life scores. In the fully adjusted model, living alone remained associated with higher loneliness prevalence (adjusted prevalence ratio 1.99, 95% confidence interval 1.13–3.50). Depressive symptoms showed the strongest associations, with adjusted prevalence ratios of 3.59 (95% confidence interval 2.27–5.68) for mild symptoms and 5.37 (95% confidence interval 2.72–10.61) for moderate symptoms, while other domains were not independently associated.

**Discussion:**

These findings suggest that emotional and social factors, particularly depressive symptoms and living alone, showed the strongest independent associations with loneliness among older women. These results provide evidence to inform the development of targeted and gender-sensitive strategies to prevent and address loneliness in later life.

## 1 Introduction

Population ageing represents one of the most significant demographic and societal challenges of the 21st century. Globally, the World Health Organization estimates that the number of people aged 60 years and older will increase by 34% between 2019 and 2030 (World Health Organization, 2021). Europe is currently the oldest region in the world, with more than 22% of its population aged 65 years and older, a proportion that continues to rise due to increased longevity and persistently low fertility rates (Eurostat, 2026).

In Spain, demographic ageing is particularly pronounced. The proportion of people aged 65 years and older is projected to increase from 20.4% to 30.5% by 2055 (Instituto Nacional de Estadística (INE), 2024). Longevity has also risen substantially, with the number of centenarians reaching 19,639 in 2022, representing a 112% increase compared with 2010. Most centenarians are women, reflecting well-documented gender differences in life expectancy (Instituto Nacional de Estadística (INE), 2022).

Within this demographic context, unwanted loneliness has emerged as an important public health concern. Its burden has been described as comparable to that of other established health risk factors and is estimated to be associated with more than 100 deaths per hour worldwide (World Health Organization, 2023). This growing recognition has led to the development of policy initiatives in several countries, including the recent approval in Spain of the State Strategic Framework on Loneliness 2026–2030 (Ministerio de Derechos Sociales, Consumo y Agenda 2030, 2026).

Loneliness has been conceptualized as a subjective and unpleasant experience that arises when individuals perceive their social relationships as insufficient in quantity or quality (Perlman and Peplau, 1981). It has also been described as a multidimensional construct comprising social loneliness, related to the absence of a broader social network, and emotional loneliness, associated with the lack of an intimate attachment figure (Weiss, 1973; Fierloos et al., 2021). From a social epidemiological perspective, loneliness is not only an individual experience but also a socially patterned condition shaped by the social and structural circumstances in which people age.

A growing body of research has documented the negative impact of loneliness on health and wellbeing. Evidence consistently shows that loneliness is associated with poorer mental and physical health and reduced quality of life, particularly among older adults (Kotwal et al., 2021; Majmudar et al., 2024). Several studies have reported associations with depressive symptoms, anxiety, cognitive impairment, and suicide risk (Domènech-Abella et al., 2017; de Maio Nascimento et al., 2024). Loneliness has also been linked to adverse physical outcomes, including frailty, multimorbidity, sleep disturbances, hypertension, cardiovascular disease, increased use of healthcare services, and higher all-cause mortality (Gené-Badia et al., 2020; Lennartsson et al., 2022).

Older adults may be particularly vulnerable to loneliness due to life-course transitions and cumulative social and health-related losses, such as widowhood, living alone, retirement, reduced social networks, functional decline, and multimorbidity. In contrast, protective factors such as having a partner, close friends, or a trusted confidant are associated with lower levels of loneliness, highlighting the importance of meaningful and supportive relationships in later life (Fierloos et al., 2021; Hajek et al., 2024; Di Gessa et al., 2025). These patterns suggest that loneliness in older age is socially distributed and closely linked to differences in living conditions, social ties, and access to supportive relationships.

Beyond its mental and physical health consequences, loneliness is also a widespread social phenomenon. Although it affects individuals of all ages, it is a particularly relevant public health concern among older populations. Across countries and regions, prevalence varies considerably, with loneliness estimated to affect between 20% and 35% of the adult population (Surkalim et al., 2022; World Health Organization, 2025). Within Europe, prevalence also varies substantially, ranging from 4% in Denmark to 15% in Greece among adults aged over 50 years (Sheftel et al., 2024). This pattern is consistent with evidence showing lower prevalence in Northern Europe and higher prevalence in Southern Europe (Schroyen et al., 2023), suggesting that loneliness is shaped by broader social and contextual conditions.

Among older adults, the prevalence of severe loneliness reaches 20.8% in women and 16.3% in men, highlighting marked gender inequalities (Hajek et al., 2025). Gender therefore constitutes a key structural determinant of loneliness in later life, with women consistently showing greater vulnerability, partly due to longer life expectancy, a higher likelihood of widowhood and living alone, and fewer economic and educational resources (Miao et al., 2025). In Spain, available evidence confirms that loneliness is a frequent and significant issue among older adults, with consistently higher prevalence among women (Domènech-Abella et al., 2017; Gené-Badia et al., 2020; Ibáñez-Del Valle et al., 2022).

Given this growing evidence and its public health implications, numerous strategies and interventions have been developed to prevent and reduce unwanted loneliness among older adults. However, available evidence highlights the importance of adapting these interventions to the specific characteristics and needs of each population, reinforcing the need for contextualized studies to inform appropriate actions (Fakoya et al., 2020; Coll-Planas et al., 2024; Jiang et al., 2025).

**FIGURE 1:**
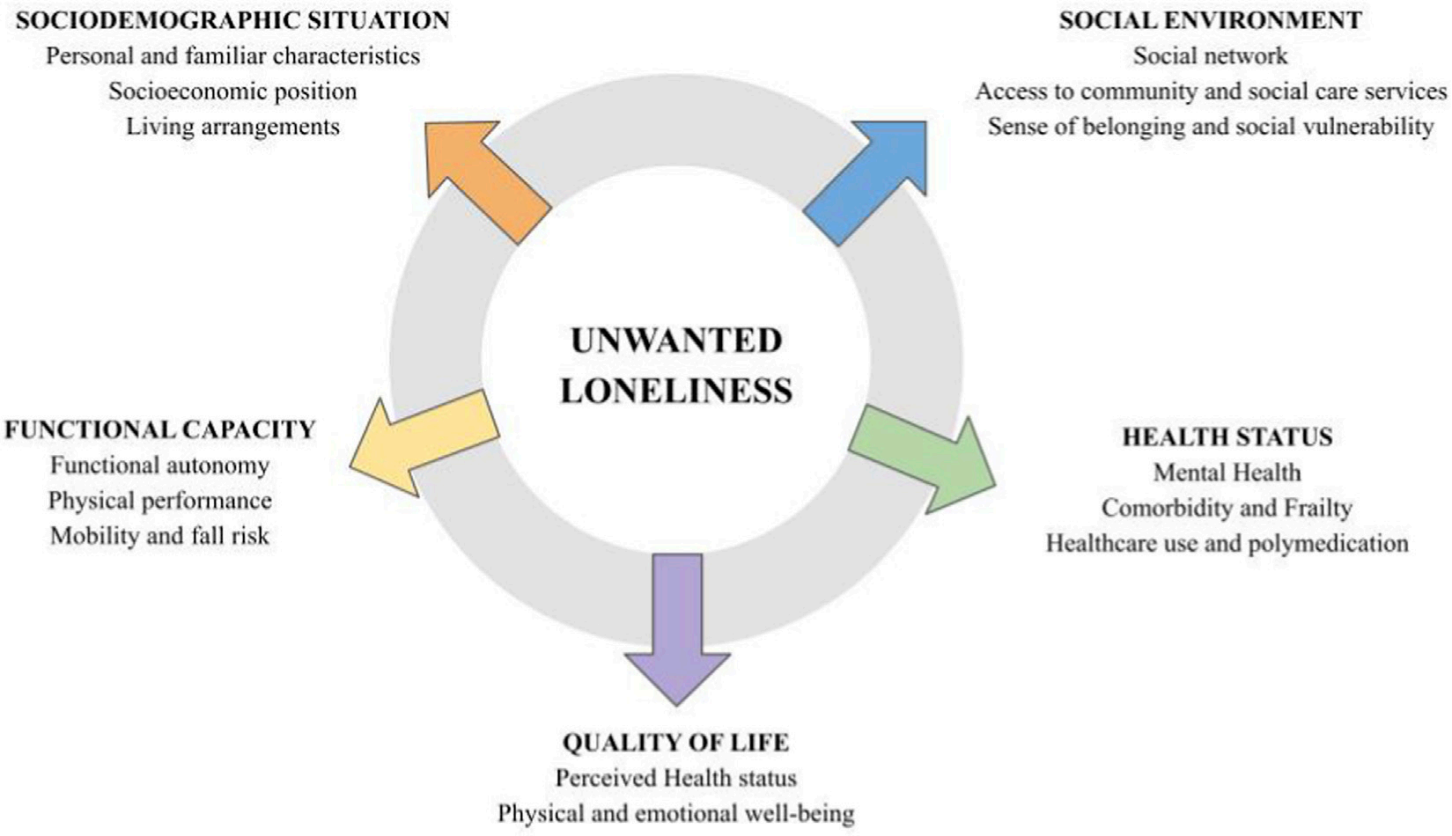
Conceptual model of unwanted loneliness in the Not Alone study. Own elaboration based on: Puyané M, Chabrera C, Camón E, Cabrera E. Uncovering the impact of loneliness in ageing populations: a comprehensive scoping review. BMC Geriatrics. 2025; 25:244.

The conceptual framework guiding this study considers unwanted loneliness as a multidimensional and socially patterned phenomenon arising from the interaction of social, health, and individual factors across the life course. Previous research has shown that loneliness in older age is associated with multiple domains, including social relationships, health conditions, functional capacity, and overall wellbeing (Domènech-Abella et al., 2017; Lennartsson et al., 2022; Majmudar et al., 2024). Based on this perspective, the model conceptualizes loneliness as the result of the interaction between five domains shaping the experience of ageing: sociodemographic conditions, social environment, functional capacity, health status, and quality of life. These domains are assumed to interact dynamically across the life course, influencing vulnerability or protection against loneliness in later life. This multidimensional approach is consistent with previous conceptual mappings of loneliness in ageing populations (Puyané et al., 2025). Figure 1 presents the conceptual framework guiding this study.

The NOT ALONE project (Aging and Loneliness: The feminization of unwanted loneliness in elderly people; how to face it) was developed as a multi-phase research program to generate evidence on the social, health, and gendered determinants of loneliness among older women and to inform targeted interventions. A specific focus on women is warranted, as loneliness in later life is shaped by gendered life-course conditions, including greater exposure to widowhood, living alone, and social and economic disadvantage. The first phase of the project, presented in this study, provides population-based evidence on the prevalence of loneliness and its associated factors among community-dwelling older women. Subsequent phases will explore women’s lived experiences of loneliness through qualitative approaches and develop predictive models to support early identification and intervention in community settings. Therefore, the aim of this study is to estimate the prevalence of loneliness among community-dwelling women aged 65 years and older and to identify the sociodemographic, social, clinical, functional, and quality-of-life factors associated with unwanted loneliness. This evidence is essential to inform the development of targeted and gender-sensitive strategies to prevent and address loneliness in later life.

## 2 Methods

### 2.1 Study design and sample

This study corresponds to the cross-sectional, population-based phase of the NOT ALONE project. The research was conducted in Mataró (Catalonia, Spain), where 11,271 women aged 65 years and older were registered in the municipal census in 2023. The target population therefore comprised community-dwelling older women.

Participants were eligible if they were able to provide informed consent. Women living in nursing homes or other long-term care institutions, those with cognitive impairment, and those with severe language barriers preventing effective participation were excluded.

A population-based random sampling strategy was used. The sampling frame was obtained from the 2023 municipal census of Mataró and included all community-dwelling women aged 65 years and older registered in the city. A computer-generated random selection procedure was applied to identify potential participants.

The required sample size was 494 participants, calculated assuming a loneliness prevalence of 30%, a 95% confidence level, and a precision of ±4%. To account for expected non-response and ineligibility, a random sample of 2,800 women was drawn from the municipal census.

Before recruitment, the list was reviewed in collaboration with the Social Welfare Department of Mataró City Council to verify eligibility. Women who were living in nursing homes or other long-term care institutions, had cognitive impairment, had severe language barriers, or no longer resided in the municipality were excluded. After this verification process, 2,314 women were considered eligible for telephone contact. During the initial telephone contact, a small number of additional women were found to be ineligible because the municipal records were not fully up to date (e.g., they no longer resided in the city or had language barriers that had not been previously identified) and were therefore excluded. To assess potential nonresponse bias, the available census characteristics (age and living arrangements) were compared between participants and eligible nonparticipants.

Eligible women were contacted by telephone by trained members of the research team to confirm eligibility, provide information about the study, and invite participation. Women who agreed to participate were scheduled for a face-to-face interview conducted in their homes. Written informed consent was obtained before data collection.

**FIGURE 2:**
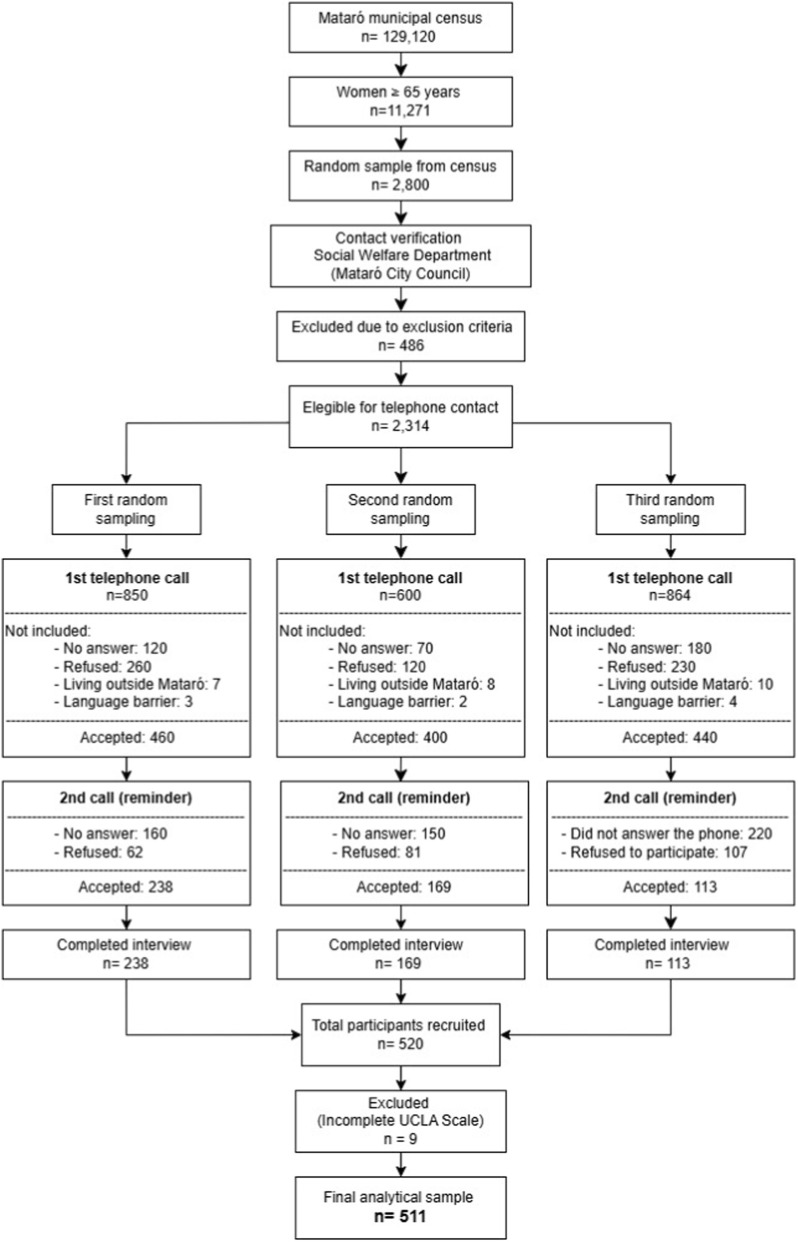
Flowchart of participant selection and recruitment process.

A total of 520 women agreed to participate and completed the interview. After excluding nine participants with incomplete UCLA Loneliness Scale data, the final analytical sample comprised 511 women. The participant selection and recruitment process is presented in Figure 2.

Data was collected between February 2024 and June 2025 through face-to-face interviews conducted in participants’ homes by trained health and social care professionals. Study data were collected and managed using Research Electronic Data Capture (REDCap) electronic data capture tools hosted at the study institution (Harris et al., 2019; RRID: SCR_003445).

### 2.2 Measures

#### 2.2.1 Primary outcome

Loneliness was assessed using the 10-item Spanish version of the UCLA Loneliness Scale (Version 3) (Russell, 1996; Velarde-Mayol et al., 2016). Total scores range from 10 to 40, with higher scores indicating lower levels of loneliness. Based on established cut-off points, scores were categorized as severe loneliness (≤20), moderate loneliness (21–30), and low or no loneliness (31–40). For the analysis, moderate and severe loneliness were combined into a dichotomous variable (loneliness vs. no loneliness). The UCLA loneliness score was dichotomized for the primary analysis to identify individuals with clinically meaningful levels of loneliness and to facilitate interpretation of associations from a public health perspective. Although loneliness is inherently a dimensional construct, dichotomized approaches using the UCLA Loneliness Scale are frequently applied in epidemiological and public health research to estimate prevalence and identify clinically meaningful loneliness (Gosling et al., 2024).

#### 2.2.2 Explanatory variables

Explanatory variables were selected *a priori* according to the study conceptual framework, which conceptualizes loneliness as a multidimensional phenomenon shaped by the interaction of social, health, and individual factors across the life course. Variables were grouped into five domains: sociodemographic conditions, social environment, health status, functional capacity, and quality of life (Figure 1).

The sociodemographic domain included age, country of birth, educational level, marital status, household composition, and monthly household income. The social domain included perceived community belonging, participation in senior associations, awareness of social resources, telecare use, receipt of home health or social care services, and social vulnerability assessed using the TSO Social Risk Assessment Scale (Giménez-Bertomeu et al., 2020).

The health status domain included depressive symptoms measured with the 17-item Hamilton Depression Rating Scale (Hamilton, 1960; Ramos-Brieva and Cordero-Villafafila, 1988), comorbidity burden assessed with the Charlson Comorbidity Index (Charlson et al., 1987), and frailty measured with the Clinical Frailty Scale (Rockwood et al., 2005). Functional capacity was assessed using the Barthel Index (Mahoney and Barthel, 1965; Cid-Ruzafa and Damián-Moreno, 1997) and the Short Physical Performance Battery (Guralnik et al., 1994), while fall risk was evaluated with the Downton fall Risk Index (Downton, 1993; Aranda-Gallardo et al., 2015). Health-related quality of life was assessed using the EQ-5D-5L, including the EQ visual analogue scale (EQ-VAS) (EuroQol Group, 1990; Ramos-Goñi et al., 2018).

All instruments used in the study were established measures with published validation studies, including Spanish validation where available. Additional information on instrument characteristics, scoring, categorization, and validation references is provided in Supplementary Table 1.

### 2.3 Statistical analysis

Sample characteristics were summarized using frequencies and percentages for categorical variables and medians with interquartile ranges (IQRs) for continuous variables. Loneliness was analyzed as a binary outcome (moderate/severe vs. low/no loneliness) because of the low frequency of severe loneliness.

Crude associations between explanatory variables and loneliness were examined using generalized linear models with Poisson distribution, log link, and robust variance to estimate prevalence ratios (PRs) and 95% confidence intervals (95% CIs) (Barros and Hirakata, 2003).

For bivariate analyses, missing categories were retained for variables with the highest proportion of missing data, namely, the Hamilton Depression Rating Scale (3.1%) and the Short Physical Performance Battery (2.3%); missingness in all other variables was below 1.3%. Multivariable analyses were based on complete-case data.

Hierarchical multivariable models were fitted to estimate adjusted prevalence ratios (aPRs), entering variables sequentially according to the predefined conceptual domains: sociodemographic conditions, social environment, health status, functional capacity, and quality of life. Collinearity was assessed using variance inflation factors (VIFs), and model fit using the log-likelihood, Akaike information criterion (AIC), and Bayesian information criterion (BIC). Multicollinearity was assessed using variance inflation factors (VIF). Individual VIF values for all variables included in the final multivariable model (Model 5) are presented in Supplementary Table S3. VIF values ranged from 1.11 to 2.29, indicating no evidence of problematic multicollinearity.

As a sensitivity analysis, the hierarchical models were repeated using the continuous UCLA loneliness score (range 10–40) as the outcome variable, applying linear regression models with robust standard errors and including the same covariates as in the primary analyses. Model assumptions were assessed by examining residuals to evaluate the adequacy of the final models. Continuous variables were scaled to improve interpretability, with the EQ-5D index expressed per 0.1-point increase and the EQ-VAS per 10-point increase. Analyses were performed using Stata version 15.1 (StataCorp, College Station, TX, United States).

### 2.4 Ethics approval and informed consent

The study was conducted in accordance with the Declaration of Helsinki and applicable ethical and data protection regulations. Ethical approval was obtained from the Ethics Committee of Fundació Tecnocampus Mataró-Maresme (code 6/2023; approval date: 31 January 2024) and from the Clinical Research Ethics Committee of the IDIAP Jordi Gol (CEIm IDIAP Jordi Gol; code 24/074-P; approval date: 29 May 2024). Written informed consent was obtained from all participants prior to data collection. Participants’ confidentiality and privacy were ensured throughout the study, and data were handled in accordance with applicable data protection regulations.

### 2.5 Reporting guidelines

This study was reported in accordance with the Strengthening the Reporting of Observational Studies in Epidemiology (STROBE) guidelines for cross-sectional studies (von Elm et al., 2007). The complete STROBE checklist is provided as supplementary material.

## 3 Results

### 3.1 Sample characteristics

To assess potential selection bias, the available census characteristics were compared between participants and eligible nonparticipants. Both groups showed very similar demographic profiles. Mean age was 77.2 years among participants and 77.1 years among nonparticipants, and the distributions of age groups and living arrangements were comparable. These findings suggest that 520 participants were broadly representative of the eligible population with respect to the available demographic characteristics.

A total of 511 community-dwelling women aged 65 years and older were finally included in the analysis. The largest age group was 66–74 years (44.4%). Most participants were born in Spain (91.2%) and had a basic level of education (41.3%). Nearly half were married (46.5%), 35.9% were widowed, and 35.6% lived alone. Regarding household income, 35.6% reported a monthly income below EUR 1,000, while 55.6% reported an income between EUR 1,000 and EUR 2,500.

In the social domain, 69.3% reported feeling part of their community or neighborhood. Participation in senior associations or groups was limited: 61.7% were not involved, 11.2% reported that they would like to participate, and 27.1% were currently participating. According to the TSO Social Risk Assessment Scale, 12.3% were at risk of social problems and 3.4% had an established social problem, indicating that 15.7% of the sample presented some degree of social vulnerability.

Clinically, 24.3% of participants had mild depressive symptoms and 3.5% had moderate depressive symptoms, while most had no comorbidity according to the Charlson Comorbidity Index (91.8%). In the functional domain, 61.2% were independent according to the Barthel Index, 48.3% were classified as frail according to the Short Physical Performance Battery, and 23.7% were at high risk of falls. Median quality-of-life scores were 0.841 [IQR 0.687–0.922] for the EQ-5D index and 80 [IQR 63–87] for the EQ-VAS.

Overall, the sample reflected a predominantly community-dwelling and relatively autonomous population of older women, although important social, emotional, and functional vulnerabilities were identified. More than one-third of participants lived alone or reported low household income, while a substantial proportion presented frailty, depressive symptoms, or risk of social problems. These findings illustrate the heterogeneity of the sample and the coexistence of social, clinical, and functional vulnerabilities potentially related to loneliness in later life. Full sample characteristics are presented in Table 1.

**TABLE 1:** Sample characteristics of community-dwelling women aged 65 years and older in the NOT ALONE study, Spain, 2024–2025.

Domain/Category		n	%
Sociodemographic domain			
Age group		​	​
66–74 years		227	44.4
75–84 years		192	37.6
≥85 years		92	18.0
Country of birth		​	​
Spain		466	91.2
Other		45	8.8
Educational level		​	​
No formal education		159	31.1
Basic education (compulsory)		211	41.3
Secondary/higher education		141	27.6
Marital status		​	​
Married		236	46.5
Widowed		182	35.9
Divorced/separated/single		89	17.6
Household composition		​	​
Living alone		182	35.6
Living with one person		234	45.8
Living with two or more people		95	18.6
Monthly household income (approx.)		​	​
<EUR 1,000		180	35.6
EUR 1,000-EUR 2,500		281	55.6
>EUR 2,500		44	8.7
Social domain			
Sense of belonging to community/neighbourhood		​	​
No		118	23.1
Partially		38	7.4
Yes		354	69.3
Participation in senior associations/groups		​	​
No		315	61.7
No, but would like to participate		57	11.2
Yes		138	27.1
Awareness of community resources		​	​
No		220	43.1
Yes		291	56.9
Telecare service at home		​	​
No		386	75.7
Yes		124	24.3
Home health or social care services		​	​
No		430	84.3
Yes		80	15.7
Social risk assessment scale (TSO)		​	​
No social problem		427	84.4
Risk of social problem		62	12.3
Established social problem		17	3.4
Clinical domain			
Hamilton depression rating scale (HDRS)		​	​
No depression		353	69.1
Mild depression		124	24.3
Moderate depression		18	3.5
Missing		16	3.1
Charlson comorbidity index		​	​
No comorbidity		469	91.8
Low comorbidity		25	4.9
High comorbidity		17	3.3
Clinical frailty scale (CFS)		​	​
Not frail		395	77.9
Vulnerable		55	10.9
Frail		57	11.2
Functional domain			
Barthel index		​	​
Independent		311	61.2
Mild dependency		70	13.8
Moderate dependency		102	20.1
Severe/total dependency		25	4.9
Short physical performance battery (SPPB)		​	​
Not frail		252	49.3
Frail		247	48.3
Missing		12	2.3
Downton fall risk index		​	​
Low risk of falls		384	75.1
High risk of falls		121	23.7
Quality-of-life domain			
EQ-5D index	Median [IQR]	​	0.841 [0.687–0.922]
EQ-VAS	Median [IQR]	​	80 [63–87]

Percentages were calculated using available data. Missingness was 3.1% for HDRS, 2.3% for SPPB, and <1.3% for all other variables.

### 3.2 Prevalence of loneliness and crude associations

The overall prevalence of loneliness (moderate or severe) measured with the UCLA Loneliness Scale was 18.6% (95/511). Loneliness prevalence showed marked differences across social, clinical, and functional domains, with the highest prevalence observed among women with established social problems (70.6%), moderate depressive symptoms (61.1%), severe or total dependency (36.0%), and high risk of falls (35.5%).

In crude analyses, loneliness was more frequent among older women, reaching 26.1% among those aged 85 years and older (PR 1.74; 95% CI 1.10–2.77), and among widowed (24.7%; PR 1.95; 95% CI 1.28–2.96) and divorced/separated/single women (22.5%; PR 1.77; 95% CI 1.06–2.95), compared with married women. Household composition showed one of the strongest associations: women living alone had more than twice the prevalence of loneliness compared with those living with one person (26.9% vs. 10.7%; PR 2.52; 95% CI 1.62–3.92). Lower household income was also associated with higher loneliness prevalence.

Clear differences were also observed across social indicators. Women who did not feel part of their community or neighborhood had a higher prevalence of loneliness than those who did (25.4% vs. 16.4%; PR 1.55; 95% CI 1.05–2.29). Those who reported that they would like to participate in senior associations but were not currently involved also showed a higher prevalence of loneliness than current participants (28.1% vs. 13.0%; PR 2.15; 95% CI 1.18–3.92). Social vulnerability showed the strongest gradient within this domain: loneliness prevalence was 14.3% among women with no social problem, 32.3% among those at risk of social problems, and 70.6% among those with an established social problem (PR 4.94; 95% CI 3.36–7.26).

Clinical variables showed the strongest crude associations. Compared with women without depressive symptoms, those with mild depression had 4.50 times the prevalence of loneliness (95% CI 3.01–6.72), while those with moderate depression had 6.96 times the prevalence (95% CI 4.22–11.47). Higher loneliness prevalence was also observed among women with mild dependency (PR 1.67; 95% CI 1.03–2.68) and severe/total dependency (PR 2.33; 95% CI 1.30–4.18) according to the Barthel Index, among those classified as frail by the SPPB (PR 2.61; 95% CI 1.70–4.01), and among those at high risk of falls (PR 2.68; 95% CI 1.88–3.80). Higher EQ-5D and EQ-VAS scores were inversely associated with loneliness (EQ-5D PR 0.85 per 0.1-point increase, 95% CI 0.81–0.89; EQ-VAS PR 0.98 per 10-point increase, 95% CI 0.97–0.98).

Overall, the crude analyses showed that loneliness was consistently associated with social vulnerability, depressive symptoms, poorer functional status, and lower health-related quality of life. Full crude associations are presented in Table 2.

**TABLE 2:** Prevalence of loneliness (moderate/severe) and crude prevalence ratios according to selected variables. NOT ALONE study, Spain, 2024–2025.

Domain/Category		N	n	Prevalence (%)	PR	95% CI	p
Total		511	95	18.6	​	​	​
Sociodemographic domain							
Age group		​	​	​	​	​	​
66–74 years		227	34	15.0	1.00	​	​
75–84 years		192	37	19.3	1.29	0.84–1.97	0.245
85+ years		92	24	26.1	1.74	1.10–2.77	0.019
Marital status		​	​	​	​	​	​
Married		236	30	12.7	1.00	​	​
Widowed		182	45	24.7	1.95	1.28–2.96	0.002
Divorced/separated/single		89	20	22.5	1.77	1.06–2.95	0.029
Household composition		​	​	​	​	​	​
Living alone		182	49	26.9	2.52	1.62–3.92	<0.001
Living with one person		234	25	10.7	1.00	​	​
Living with two or more people		95	21	22.1	2.07	1.22–3.51	0.007
Monthly household income (approx.)		​	​	​	​	​	​
Less than EUR 1,000		180	46	25.6	1.94	1.31–2.87	0.001
EUR 1,000–2,500		281	37	13.2	1.00	​	​
More than EUR 2,500		44	8	18.2	1.38	0.69–2.77	0.363
Social domain							
Sense of belonging to an active community/neighbourhood		​	​	​	​	​	​
No		118	30	25.4	1.55	1.05–2.29	0.027
Partially		38	6	15.8	0.96	0.45–2.09	0.925
Yes		354	58	16.4	1.00	​	​
Participation in senior associations/groups		​	​	​	​	​	​
No		315	60	19.1	1.46	0.90–2.38	0.128
No, but would like to participate		57	16	28.1	2.15	1.18–3.92	0.012
Yes		138	18	13.0	1.00	​	​
Social risk assessment (TSO)		​	​	​	​	​	​
No social problem		427	61	14.3	1.00	​	​
Risk of social problem		62	20	32.3	2.26	1.47–3.47	<0.001
Established social problem		17	12	70.6	4.94	3.36–7.26	<0.001
Clinical domain							
Hamilton depression rating scale (HDRS)		​	​	​	​	​	​
No depression		353	31	8.8	1.00	​	​
Mild depression		124	49	39.5	4.50	3.01–6.72	<0.001
Moderate depression		18	11	61.1	6.96	4.22–11.47	<0.001
Functional domain							
Barthel index		​	​	​	​	​	​
Independent		311	48	15.4	1.00	​	​
Mild dependency		70	18	25.7	1.67	1.03–2.68	0.036
Moderate dependency		102	18	17.7	1.14	0.70–1.87	0.595
Severe/total dependency		25	9	36.0	2.33	1.30–4.18	0.005
Short physical performance battery (SPPB)		​	​	​	​	​	​
Not frail		252	25	9.9	1.00	​	​
Frail		247	64	25.9	2.61	1.70–4.01	<0.001
Downton fall risk index		​	​	​	​	​	​
Low risk of falls		384	51	13.3	1.00	​	​
High risk of falls		121	43	35.5	2.68	1.88–3.80	<0.001
Quality-of-life domain							
EQ-5D (per 0.1-point increase)	No loneliness: 0.899 [0.721–1.000]; loneliness: 0.726 [0.551–0.841]	​	​	​	0.85	0.81–0.89	<0.001
EQ-VAS (per 10-point increase)	No loneliness: 80 [70–90]; loneliness: 70 [50–80]	​	​	​	0.98	0.97–0.98	<0.001

Models were estimated using Poisson regression with robust variance. Values are crude prevalence ratios (PRs) with 95% confidence intervals (CIs). Missing categories are shown only for the Hamilton Depression Rating Scale and the Short Physical Performance Battery because missingness exceeded 2%; missingness for all remaining variables was <1.3%.

### 3.3 Multivariable models of loneliness

Hierarchical multivariable Poisson regression models with robust variance were fitted to identify factors independently associated with loneliness. Model fit improved progressively with the sequential inclusion of domains, with the lowest AIC observed for the fully adjusted model (Model 5: AIC 451.64), which was estimated using 502 complete cases.

Within the sociodemographic domain, household composition remained the most consistent correlation of loneliness across models. In the fully adjusted model, women living alone had nearly twice the prevalence of loneliness compared with those living with one person (aPR 1.99; 95% CI 1.13–3.50). Living with two or more people showed a weaker and non-significant association in the final model (aPR 1.54; 95% CI 0.91–2.61). By contrast, age, marital status, and household income were no longer significantly associated with loneliness after full adjustment.

Within the social domain, the presence of social problems according to the TSO scale was associated with higher loneliness prevalence after adjustment for sociodemographic variables, particularly for women with an established social problem (Model 2: aPR 3.12; 95% CI 1.88–5.15). However, this association attenuated after the inclusion of clinical, functional, and quality-of-life variables and was no longer statistically significant in the final model (Model 5: aPR 1.59; 95% CI 0.89–2.84). Participation in senior associations was not independently associated with loneliness in the fully adjusted model.

Clinical variables showed the strongest independent associations. Compared with women without depressive symptoms, those with mild depression had a 3.59-fold higher prevalence of loneliness (95% CI 2.27–5.68), while those with moderate depression had a 5.37-fold higher prevalence (95% CI 2.72–10.61) in the fully adjusted model.

Most functional and quality-of-life indicators were not independently associated with loneliness in the final model. An exception was moderate dependency on the Barthel Index, which showed a lower prevalence of loneliness compared with full independence (aPR 0.42; 95% CI 0.24–0.74). Mild dependency, severe/total dependency, EQ-5D, and EQ-VAS were not significantly associated with loneliness after full adjustment. Full model estimates are presented in Table 3.

**TABLE 3:** Hierarchical multivariable Poisson regression models for loneliness (moderate/severe) measured with the UCLA Loneliness Scale. NOT ALONE study, Spain, 2024–2025.

Variable	Model 1 aPR (95% CI)	Model 2 aPR (95% CI)	Model 3 aPR (95% CI)	Model 4 aPR (95% CI)	Model 5 aPR (95% CI)
Sociodemographic domain					
*Age group (ref: 66–74 years)*	​	​	​	​	​
75–84 years	1.23 [0.80–1.88]	1.16 [0.75–1.79]	1.08 [0.70–1.57]	1.14 [0.74–1.76]	1.09 [0.71–1.69]
≥85 years	1.36 [0.82–2.26]	1.31 [0.78–2.21]	1.12 [0.65–1.94]	1.20 [0.68–2.12]	1.14 [0.64–2.03]
*Marital status (ref: Married)*	​	​	​	​	​
Widowed	1.15 [0.66–2.02]	1.01 [0.57–1.80]	1.08 [0.64–1.83]	1.10 [0.67–1.80]	1.16 [0.70–1.93]
Divorced/separated/single	1.25 [0.69–2.28]	1.02 [0.54–1.92]	1.17 [0.62–2.20]	1.17 [0.63–2.16]	1.30 [0.68–2.49]
*Household composition (ref: Living with one person)*	​	​	​	​	​
Living alone	1.92 [1.12–3.28]	1.93 [1.09–3.44]	1.94 [1.10–3.42]	2.11 [1.22–3.64]	1.99 [1.13–3.50]
Living with two or more people	1.90 [1.13–3.19]	1.85 [1.11–3.08]	1.61 [0.95–2.72]	1.59 [0.94–2.68]	1.54 [0.91–2.61]
*Monthly household income (ref: €1,000–€2,500)*	​	​	​	​	​
<€1,000	1.74 [1.17–2.59]	1.39 [0.92–2.09]	1.11 [0.73–1.67]	1.09 [0.70–1.68]	1.02 [0.65–1.62]
>€2,500	1.48 [0.76–2.87]	1.57 [0.81–3.05]	1.45 [0.76–2.77]	1.36 [0.73–2.56]	1.31 [0.62–2.80]
Social domain					
*Participation in senior associations/groups (ref: Yes)*	​	​	​	​	​
No	—	1.37 [0.85–2.20]	1.40 [0.89–2.19]	1.53 [0.97–2.42]	1.55 [0.97–2.49]
No, but would like to participate	—	1.81 [1.03–3.19]	1.46 [0.86–2.50]	1.51 [0.90–2.54]	1.35 [0.76–2.40]
*Social Risk Assessment Scale (TSO) (ref: No social problem)*	​	​	​	​	​
Risk of social problem	—	1.71 [1.07–2.73]	1.39 [0.90–2.16]	1.60 [1.01–2.53]	1.50 [0.93–2.41]
Established social problem	—	3.12 [1.88–5.15]	2.06 [1.35–3.15]	2.08 [1.27–3.41]	1.59 [0.89–2.84]
Clinical domain					
*Hamilton Depression Rating Scale (HDRS) (ref: No depression)*	​	​	​	​	​
Mild depression	—	—	3.83 [2.51–5.86]	4.00 [2.60–6.16]	3.59 [2.27–5.68]
Moderate depression	—	—	4.94 [2.62–9.32]	6.24 [3.22–12.09]	5.37 [2.72–10.61]
Functional domain					
*Barthel Index (ref: Independent)*	​	​	​	​	​
Mild dependency	—	—	—	1.34 [0.82–2.20]	1.39 [0.85–2.28]
Moderate dependency	—	—	—	0.51 [0.32–0.80]	0.42 [0.24–0.74]
Severe/total dependency	—	—	—	0.96 [0.50–1.83]	0.64 [0.27–1.54]
Quality-of-life domain					
*Health-related quality of life (EQ-5D), per 0.10-point increase*	—	—	—	—	0.96 [0.88–1.04]
*EQ-VAS, per 10-point increase*	—	—	—	—	0.999 [0.998–1.000]
Model fit					
N	511	511	511	511	502
Log-likelihood	−240.40	−232.40	−214.01	−208.26	−200.82
AIC	502.81	496.80	468.02	464.52	451.64
BIC	549.41	564.58	552.75	566.20	557.10

Poisson regression with robust variance. Values are aPRs, with 95% CIs. Model 1: sociodemographic; Model 2: + social; Model 3: + clinical; Model 4: + functional; Model 5: + quality of life. Reference categories: age 66–74 years, married, living with one person, monthly household income €1,000–€2,500, participation in senior associations/groups, no social problem, no depression, and independent functional status where applicable. Model 5 used complete-case data. Lower AIC, and BIC, indicate better fit.

Sensitivity analyses using the continuous UCLA loneliness score confirmed the robustness of the main findings, as the same factors were associated with loneliness in the same direction and with similar levels of statistical significance. Non-participation in associations and the presence of a social problem, which showed borderline significance in the primary analysis, reached statistical significance when using the continuous outcome, likely reflecting the greater statistical power of this approach. Inspection of residuals did not reveal substantial deviations from model assumptions. Detailed results are presented in Supplementary Table 2.

## 4 Discussion

This study aimed to estimate the prevalence of loneliness and to identify the factors associated with it among community-dwelling women aged 65 years and older. The focus on women reflects not only a demographic category, but also the influence of gendered life-course conditions that may shape vulnerability to loneliness in later life.

Two main findings stand out. First, 18.6% of community-dwelling older women reported moderate or severe loneliness, confirming that loneliness represents an important public health concern in later life. Second, loneliness was most strongly associated with living alone and depressive symptoms, whereas the associations observed for social vulnerability, functional status, and health-related quality of life were attenuated after adjustment for the other domains.

Overall, the findings suggest that loneliness in older women is associated with living conditions, emotional wellbeing, and the social contexts in which ageing takes place. These results may help inform the development of targeted and gender-sensitive strategies to prevent and address loneliness in later life.

The prevalence found in this study is within the range reported in Spain and other high-income settings, although it is lower than that described in some national and international studies (Domènech-Abella et al., 2017; Gené-Badia et al., 2020; Ibáñez-Del Valle et al., 2022; Schroyen et al., 2023; Hajek et al., 2025). This variation is consistent with previous reviews showing that loneliness estimates differ widely according to the characteristics of the population, the age structure, the setting, and the instruments used (Surkalim et al., 2022; Stegen et al., 2024). In our study, the comparatively lower prevalence may partly reflect the profile of the sample, which was largely functionally independent, had low levels of comorbidity, and reported relatively favorable quality-of-life scores. It may also reflect some selection of healthier or more socially engaged women, given that participation required accepting a face-to-face home interview.

Living arrangements emerged as one of the clearest correlations of loneliness. Women living alone showed nearly twice the adjusted prevalence of loneliness (aPR 1.99; 95% CI 1.13–3.50) compared with those living with one person. This finding is in line with previous studies showing that living alone remains a relevant marker of vulnerability in later life, particularly among women, whose longer life expectancy increases the likelihood of widowhood and prolonged solo living (Martín Roncero and González-Rábago, 2021; Inoue et al., 2025; Di Gessa et al., 2025). However, our findings suggest that the relationship between living alone and loneliness cannot be explained solely by household composition. Other factors identified in this study, including depressive symptoms, social vulnerability, reduced community belonging, and limited social participation, may help contextualize the observed association between living arrangements and loneliness.

A recent study conducted in rural areas in Spain also examined unwanted loneliness among older adults, reporting a higher prevalence and identifying living alone and low engagement in daily and social activities as relevant correlates (Yurrebaso et al., 2026). At the same time, our results reinforce an important distinction: living alone is not the same as feeling lonely. Some women living alone did not report loneliness, while some women living with others did. This supports the idea that loneliness depends not only on household composition but also on the meaning, quality, and reliability of social relationships.

This point is especially relevant when considering the social dimension of loneliness. In our study, women who did not feel part of their community or neighborhood, those who wished to participate but were not doing so, and those in situations of greater social vulnerability showed higher levels of loneliness. Although these associations weakened after accounting for emotional and health-related factors, they should not be dismissed as unimportant. Rather, they suggest that the observed association between social vulnerability and loneliness overlaps with emotional distress, reduced sense of belonging, and perceptions that available relationships are insufficient. In this sense, the findings support a social epidemiological reading of loneliness as a socially situated experience, shaped by both structural conditions and subjective social experience (Barjaková et al., 2023).

The results also highlight the potential relevance of protective social factors. Feeling part of a community and participating in social groups were associated with lower loneliness in the crude analyses, although these associations were attenuated and no longer remained independently associated after adjustment for the other domains. Nevertheless, these findings remain informative, suggesting that supportive networks, community belonging, and opportunities for meaningful participation may still represent important dimensions of social environments associated with lower loneliness, even if their associations overlap with other psychosocial factors. This interpretation is consistent with previous studies indicating that the quality and emotional significance of social relationships may be more important than the mere presence of social contacts or formal participation (Schroyen et al., 2023; Hajek et al., 2024; Di Gessa et al., 2025). These findings may also help to interpret the geographical variation reported across Europe, where differences in social norms, family structures, and welfare systems are associated with the availability and meaning of social relationships. Together, these findings support the importance of strengthening community environments, not only as places where services are delivered, but also as settings that facilitate meaningful social connections.

Depressive symptoms showed the strongest and most consistent association with loneliness. In our study, women with mild and moderate depressive symptoms showed 3.6-fold and 5.4-fold higher adjusted prevalence of loneliness, respectively. This finding is consistent with a large body of literature showing a close relationship between loneliness and depression in later life (Domènech-Abella et al., 2017; Ibáñez-Del Valle et al., 2022; de Maio Nascimento et al., 2024; Inoue et al., 2025).

Although the direction of this relationship cannot be established in a cross-sectional study, previous research suggests that loneliness and depressive symptoms may reinforce one another over time. Even without being able to establish temporality, our findings indicate that emotional wellbeing is central to understanding loneliness in older women and should not be treated as a secondary issue.

By contrast, the role of functional status and health-related quality of life appeared less direct. Frailty, poorer physical performance, fall risk, and lower health-related quality-of-life scores were associated with loneliness in the crude analyses, but these associations were attenuated after adjustment for social and emotional factors. These findings suggest that physical vulnerability may be less strongly associated with loneliness when considered independently than when viewed within a broader context of social and emotional vulnerability. Poorer health may coexist with loneliness, but the association with loneliness appears to be more closely related to how health problems are associated with autonomy, participation, and emotional wellbeing than to health status alone. The inverse association observed for moderate dependency on the Barthel Index should therefore be interpreted with caution. Although individual variance inflation factors did not indicate problematic multicollinearity (all VIF values were <2.5; Supplementary Table S3), the possibility that this counterintuitive association reflects a statistical artefact related to shared variance among correlated determinants cannot be completely excluded. Accordingly, the inverse association observed for moderate functional dependency may reflect a suppression effect or residual confounding arising from adjustment for correlated variables across sociodemographic, social, and clinical domains, rather than a true protective association. Nevertheless, it is also possible that women with moderate dependency receive greater formal or informal support, including family accompaniment, home care services, telecare, or increased social contact related to caregiving arrangements, which could partially mitigate feelings of loneliness. In addition, the relatively small number of women with severe or total dependency may have contributed to instability in the estimates for the most dependent category. Therefore, this result should not be interpreted as evidence of a protective effect of moderate functional dependency, but rather as an indication of the complexity of the relationships between functional dependency, psychosocial factors, and loneliness.

Taken together, these findings indicate that loneliness in older women is associated with the intersection of gendered life-course conditions, living arrangements, emotional distress, and the availability of meaningful social connections. Rather, loneliness appears to be associated with the intersection of gendered life-course conditions, living arrangements, emotional distress, and the availability of meaningful social connection. This view is consistent with evidence showing that loneliness in older age is socially patterned through intersecting forms of inequality rather than single risk factors alone (Gustafsson et al., 2022), and with multidimensional approaches that emphasize the interplay between structural conditions, social relationships, and psychological processes across the life course (Weiss, 1973; Puyané et al., 2025).

These findings have potential implication for practice. Efforts to address loneliness in older women should not focus only on identifying who lives alone or who has poorer health. They should also strengthen the social and community environments in which later life unfolds. Community-based approaches, accessible opportunities for participation, and actions that reinforce trusted and meaningful relationships may be as important as individual-level support. At the same time, women reporting depressive symptoms represent a group with particularly high levels of loneliness and may benefit from specific attention in prevention and intervention strategies. A more effective response to loneliness will therefore require combining emotional support, social care, and stronger community contexts.

## 5 Limitations

The findings of this study should be interpreted considering several limitations. First, the cross-sectional design precludes causal inference, particularly regarding the relationship between loneliness and depressive symptoms. Second, participation required a home interview, and institutionalized women and those with cognitive impairment were excluded. Although comparison of the available census characteristics showed that participants and eligible nonparticipants had very similar age distributions and living arrangements, suggesting limited selection bias with respect to these demographic characteristics, participation was voluntary. Therefore, differences in unmeasured characteristics, such as health status, social participation, or loneliness, cannot be ruled out. Consequently, some degree of nonresponse bias may remain, and the generalizability of the findings should be interpreted with appropriate caution Finally, some dimensions relevant to loneliness, including relationship quality, life-course experiences, and the meaning attached to social connection, were not examined in depth. As the study included only women, the findings cannot be directly generalized to older men or gender-diverse older adults.

## 6 Strengths

This study has several important strengths. It is based on a population-based sample of community-dwelling women aged 65 years and older, providing robust epidemiological evidence on loneliness in a population group that remains underrepresented in the literature despite being particularly exposed to gendered vulnerabilities in later life. The use of widely recognized and validated instruments to assess loneliness, depressive symptoms, frailty, functional status, and quality of life enhances the reliability and comparability of the findings. In addition, the hierarchical modelling strategy offers insight into how social, emotional, and health-related factors overlap in shaping the experience of loneliness.

## 7 Implications for practice and policy

These findings have important implications for practice and policy. They highlight the need to strengthen the early identification of loneliness among older women in community and primary care settings, particularly among those living alone, experiencing social vulnerability, or presenting depressive symptoms. The results also suggest that responses to loneliness should move beyond increasing opportunities for participation and explicitly address emotional wellbeing, the quality of social relationships, and barriers to meaningful engagement in community life.

At a systems level, the findings support the development of integrated, community-based approaches that combine health and social care, as fragmented responses are unlikely to adequately address the multidimensional nature of loneliness. In addition, these results provide a foundation for future work aimed at developing predictive tools to support the early identification and targeted support of older women at risk of loneliness in community settings.

## 8 Conclusion

Loneliness among community-dwelling older women appears to be more strongly associated with living arrangements, emotional wellbeing, and the social contexts of later life than with physical decline. Living alone and depressive symptoms emerged as the most consistent correlations, while broader social and functional disadvantages appeared to operate more indirectly. These findings reinforce the need to understand loneliness as a multidimensional and socially embedded experience and to address it through integrated approaches that combine individual support with stronger community environments and meaningful opportunities for connection.

## Data Availability

The data supporting the conclusions of this article will be made available by the authors upon reasonable request, subject to ethical and privacy restrictions.

## References

[B1] Aranda-Gallardo M. Morales-Asencio J. M. Canca-Sánchez J. C. Morales-Fernández Á. Enríquez de Luna-Rodríguez M. Moya-Suarez A. B. (2015). Consecuencias de los errores en la traducción de cuestionarios: versión española del índice Downton. Rev. Calid. Asist. 30, 195–202. 10.1016/j.cali.2015.04.003 26068277

[B2] Barjaková M. Garnero A. d’Hombres B. (2023). Risk factors for loneliness: a literature review. Soc. Sci. Med. 334, 116163. 10.1016/j.socscimed.2023.116163 37625251 PMC10523154

[B3] Barros A. J. D. Hirakata V. N. (2003). Alternatives for logistic regression in cross-sectional studies: an empirical comparison of models that directly estimate the prevalence ratio. BMC Med. Res. Methodol. 3, 21. 10.1186/1471-2288-3-21 14567763 PMC521200

[B4] Charlson M. E. Pompei P. Ales K. L. MacKenzie C. R. (1987). A new method of classifying prognostic comorbidity in longitudinal studies: development and validation. J. Chronic Dis. 40, 373–383. 10.1016/0021-9681(87)90171-8 3558716

[B5] Cid-Ruzafa J. Damián-Moreno J. (1997). Valoración de la discapacidad física: el índice de Barthel. Rev. Esp. Salud Publica 71, 127–137. 10.1590/s1135-57271997000200004 9546856

[B6] Coll-Planas L. Carbó-Cardeña A. Jansson A. Dostálová V. Bartova A. Rautiainen L. (2024). Nature-based social interventions to address loneliness among vulnerable populations: a common study protocol for three related randomized controlled trials in Barcelona, Helsinki, and Prague within the RECETAS European project. BMC Public Health 24, 172. 10.1186/s12889-023-17547-x 38218784 PMC10787456

[B7] de Maio Nascimento M. Lampraki C. Marques A. Gouveia É. R. Adsuar J. C. Ihle A. (2024). Longitudinal cross-lagged analysis of depression, loneliness, and quality of life in 12 European countries. BMC Public Health 24, 1986. 10.1186/s12889-024-19463-0 39054451 PMC11270973

[B8] Di Gessa G. Bordone V. Arpino B. (2025). Trajectories of loneliness in later life: evidence from a 10-year English panel study. Soc. Sci. Med. 382, 117703. 10.1016/j.socscimed.2025.117703 39956740 PMC12264031

[B9] Domènech-Abella J. Lara E. Rubio-Valera M. Olaya B. Moneta M. V. Rico-Uribe L. A. (2017). Loneliness and depression in the elderly: the role of social network. Soc. Psychiatry Psychiatr. Epidemiol. 52, 381–390. 10.1007/s00127-017-1339-3 28154893

[B10] Downton J. H. (1993). Falls in the Elderly. London: Edward Arnold.

[B11] EuroQol Group (1990). EuroQol: a new facility for the measurement of health-related quality of life. Health Policy 16, 199–208. 10.1016/0168-8510(90)90421-9 10109801

[B12] Eurostat (2026). Population structure and ageing. Available online at: https://ec.europa.eu/eurostat/statistics-explained/index.php?title=Population_structure_and_ageing (Accessed March 10, 2026).

[B13] Fakoya O. A. McCorry N. K. Donnelly M. (2020). Loneliness and social isolation interventions for older adults: a scoping review of reviews. BMC Public Health 20, 129. 10.1186/s12889-020-8251-6 32054474 PMC7020371

[B14] Fierloos I. N. Tan S. S. Williams G. Alhambra-Borrás T. Koppelaar E. Bilajac L. (2021). Socio-demographic characteristics associated with emotional and social loneliness among older adults. BMC Geriatr. 21, 114. 10.1186/s12877-021-02058-4 33563228 PMC7871533

[B15] Gené-Badia J. Comice P. Belchín A. Erdozain M. Á. Cáliz L. Torres S. (2020). Perfiles de soledad y aislamiento social en población urbana. Aten. Primaria 52, 224–232. 10.1016/j.aprim.2018.09.012 30770152 PMC7118570

[B16] Giménez-Bertomeu V.-M. Guinovart-Garriga C. Rovira-Soler E. Viñas-Segalés N. (2020). La Escala de Valoración Sociofamiliar TSO. Fundamentos, descripción, validación e instrucciones de uso. Versión 1. Español. Universidad de Alicante. Available online at: http://hdl.handle.net/10045/110282 (Accessed February 20, 2026).

[B17] Gosling C. J. Colle R. Cartigny A. Jollant F. Corruble E. Frajerman A. (2024). Measuring loneliness: a head-to-head psychometric comparison of the 3- and 20-item UCLA loneliness scales. Psychol. Med. 54, 3821–3827. 10.1017/S0033291724002083 39479755 PMC11578901

[B18] Guralnik J. M. Simonsick E. M. Ferrucci L. Glynn R. J. Berkman L. F. Blazer D. G. (1994). A short physical performance battery assessing lower extremity function: association with self-reported disability and prediction of mortality and nursing home admission. J. Gerontol. 49, M85–M94. 10.1093/geronj/49.2.m85 8126356

[B19] Gustafsson P. E. Fonseca-Rodríguez O. Nilsson I. San Sebastián M. (2022). Intersectional inequalities in loneliness among older adults before and during the early phase of the COVID-19 pandemic: a total population survey in the Swedish eldercare setting. Soc. Sci. Med. 314, 115447. 10.1016/j.socscimed.2022.115447 36288648 PMC9556960

[B20] Hajek A. Gyasi R. M. König H.-H. (2024). Factors associated with loneliness among individuals aged 80 years and over: findings derived from the nationally representative “Old Age in Germany (D80+)” study. Arch. Gerontol. Geriatr. 123, 105443. 10.1016/j.archger.2024.105443 38631279

[B21] Hajek A. Volkmar A. König H.-H. (2025). Prevalence and correlates of loneliness and social isolation in the oldest old: a systematic review, meta-analysis and meta-regression. Soc. Psychiatry Psychiatr. Epidemiol. 60, 993–1015. 10.1007/s00127-023-02602-0 38102477 PMC12119783

[B22] Hamilton M. (1960). A rating scale for depression. J. Neurol. Neurosurg. Psychiatry 23, 56–62. 10.1136/jnnp.23.1.56 14399272 PMC495331

[B23] Harris P. A. Taylor R. Minor B. L. Elliott V. Fernandez M. , and REDCap Consortium (2019). The REDCap consortium: building an international community of software platform partners. J. Biomed. Inf. 95, 103208. 10.1016/j.jbi.2019.103208 31078660 PMC7254481

[B24] Ibáñez-Del Valle V. Corchón S. Zaharia G. Cauli O. (2022). Social and emotional loneliness in older community dwelling-individuals: the role of socio-demographics. Int. J. Environ. Res. Public Health 19, 16622. 10.3390/ijerph192416622 36554512 PMC9779629

[B25] Inoue Y. Hamada H. Nakatani H. Ono I. (2025). Loneliness-associated factors among older adults: focus on friendship type and number of friends. Jpn. J. Nurs. Sci. 22, e12649. 10.1111/jjns.12649 39828632 PMC11743425

[B26] Instituto Nacional de Estadística (INE) (2022). Proporción de personas mayores de cierta edad por provincia. INEbase. Available online at: https://www.ine.es/ (Accessed March 3, 2026).

[B27] Instituto Nacional de Estadística (INE) (2024). Proyecciones de población 2024–2074. Available online at: https://www.ine.es/dyngs/Prensa/PROP20242074.htm (Accessed March 3, 2026).

[B28] Jiang D. Kwok J. Y. Y. Yeung D.Y.-L. Tang V. F. Y. Choi N. G. Ho R. T. H. (2025). Six-month outcomes of layperson-delivered, telephone-based behavioural activation and mindfulness interventions on loneliness among older adults during the COVID-19 pandemic: the HEAL-HOA dual randomised controlled trial. Age Ageing 54, afaf209. 10.1093/ageing/afaf209 40748314 PMC12315544

[B29] Kotwal A. A. Cenzer I. S. Waite L. J. Covinsky K. E. Perissinotto C. M. Boscardin W. J. (2021). The epidemiology of social isolation and loneliness among older adults during the last years of life. J. Am. Geriatr. Soc. 69, 3081–3091. 10.1111/jgs.17366 34247388 PMC8595510

[B30] Lennartsson C. Rehnberg J. Dahlberg L. (2022). The association between loneliness, social isolation and all-cause mortality in a nationally representative sample of older women and men. Aging Ment. Health 26, 1821–1828. 10.1080/13607863.2021.1976723 34550832

[B31] Mahoney F. I. Barthel D. W. (1965). Functional evaluation: the Barthel index. Md. State Med. J. 14, 61–65. 14258950

[B32] Majmudar I. K. Mihalopoulos C. Abimanyi-Ochom J. Mohebbi M. Engel L. (2024). The association between loneliness with health service use and quality of life among informal carers in Australia. Soc. Sci. Med. 348, 116821. 10.1016/j.socscimed.2024.116821 38569284

[B33] Martín Roncero U. González-Rábago Y. (2021). Unwanted loneliness, health and social inequalities throughout the life cycle. Gac. Sanit. 35, 432–437. 10.1016/j.gaceta.2020.07.010 32948332

[B34] Miao M.-Y. Fang F. Lyu J.-Q. Liu Z.-Y. Wan Z.-X. Qin L.-Q. (2025). Relationships of social isolation and loneliness with healthy aging among older adults. BMC Geriatr. 25, 267. 10.1186/s12877-025-05941-6 40269703 PMC12016190

[B35] Ministerio de Derechos Sociales, Consumo y Agenda 2030 (2026). Marco Estratégico Estatal de las Soledades 2026–2030. Madrid: Gobierno de España.

[B36] Perlman D. Peplau L. A. (1981). “Toward a social psychology of loneliness,” in Personal Relationships in Disorder. Editors Duck S. Gilmour R. (Academic Press), 31–56.

[B37] Puyané M. Chabrera C. Camón E. Cabrera E. (2025). Uncovering the impact of loneliness in ageing populations: a comprehensive scoping review. BMC Geriatr. 25, 244. 10.1186/s12877-025-05846-4 40211165 PMC11984289

[B38] Ramos-Brieva J. A. Cordero-Villafafila A. (1988). A new validation of the Hamilton rating scale for depression. J. Psychiatr. Res. 22, 21–28. 10.1016/0022-3956(88)90024-6 3397906

[B39] Ramos-Goñi J. M. Craig B. M. Oppe M. Ramallo-Fariña Y. Pinto-Prades J. L. Luo N. (2018). Handling data quality issues to estimate the Spanish EQ-5D-5L value set using a hybrid interval regression approach. Value Health 21, 596–604. 10.1016/j.jval.2017.10.023 29753358

[B40] Rockwood K. Song X. MacKnight C. Bergman H. Hogan D. B. McDowell I. (2005). A global clinical measure of fitness and frailty in elderly people. CMAJ 173, 489–495. 10.1503/cmaj.050051 16129869 PMC1188185

[B41] Russell D. W. (1996). UCLA loneliness scale (version 3): reliability, validity, and factor structure. J. Pers. Assess. 66, 20–40. 10.1207/s15327752jpa6601_2 8576833

[B42] Schroyen S. Janssen N. Duffner L. A. Veenstra M. Pyrovolaki E. Salmon E. (2023). Prevalence of loneliness in older adults: a scoping review. Health Soc. Care Community 2023, 1–12. 10.1155/2023/7726692

[B43] Sheftel M. G. Margolis R. Verdery A. M. (2024). Loneliness trajectories and chronic loneliness around the world. J. Gerontol. B Psychol. Sci. Soc. Sci. 79, gbae098. 10.1093/geronb/gbae098 38814952 PMC11227000

[B44] Stegen H. Duppen D. Savieri P. Stas L. Pan H. Aartsen M. (2024). Loneliness prevalence of community-dwelling older adults and the impact of the mode of measurement, data collection, and country: a systematic review and meta-analysis. Int. Psychogeriatr. 36, 747–761. 10.1017/S1041610224000425 38525677

[B45] Surkalim D. L. Luo M. Eres R. Gebel K. van Buskirk J. Bauman A. (2022). The prevalence of loneliness across 113 countries: systematic review and meta-analysis. BMJ 376, e067068. 10.1136/bmj-2021-067068 35140066 PMC8826180

[B46] Velarde-Mayol C. Fragua-Gil S. García-de-Cecilia J. M. (2016). Validación de la escala de soledad de UCLA y perfil social en la población anciana que vive sola. Semergen 42, 177–183. 10.1016/j.semerg.2015.05.017 26187595

[B47] von Elm E. Altman D. G. Egger M. Pocock S. J. Gøtzsche P. C. Vandenbroucke J. P. (2007). The strengthening the reporting of observational studies in epidemiology (STROBE) statement: guidelines for reporting observational studies. PLoS Med. 4, e296. 10.1371/journal.pmed.0040296 17941714 PMC2020495

[B48] Weiss R. S. (1973). Loneliness: The Experience of Emotional and Social Isolation. Cambridge, MA: MIT Press.

[B49] World Health Organization (2021). Decade of healthy ageing: plan of action 2021–2030. Available online at: https://www.who.int/es/publications/m/item/decade-of-healthy-ageing-plan-of-action (Accessed January 15, 2026).

[B50] World Health Organization (2023). Loneliness and isolation: the hidden threat to global health we can no longer ignore. Available online at: https://www.who.int/es/news-room/commentaries/detail/loneliness-and-isolation-the-hidden-threat-to-global-health-we-can-no-longer-ignore (Accessed January 15, 2026).

[B51] World Health Organization (2025). From loneliness to social connection: charting a path to healthier societies. Geneva: World Health Organization. Available online at: https://www.who.int/publications/i/item/978240112360 (Accessed January 15, 2026).

[B52] Yurrebaso A. Picado-Valverde E. García-Valverde E. Guzmán-Ordaz R. (2026). Unwanted loneliness among older adults in rural areas: associated factors and guidelines for community intervention. Front. Aging 7, 1681481. 10.3389/fragi.2026.1681481 41728386 PMC12917371

