# Supplementary material for: Loneliness in older women: a multidimensional population-based study of social, health, and life-course factors
Source: Front Aging. 2026 Jul 29;7:1858726. doi: 10.3389/fragi.2026.1858726 (PMC13461583; doi:10.3389/fragi.2026.1858726)
Supplement: Supplementary file 2 [file Table2.docx]

Supplementary Material

**Supplementary Table S1. Main characteristics, scoring, categorization, and validation references for the instruments used in the study.**

| **Instrument** | **Construct assessed** | **Items / Range** | **Categorisation used in this study** | **Reliability/validity information** | **Reference** |
| --- | --- | --- | --- | --- | --- |
| **UCLA Loneliness Scale (10-item version)** | Subjective loneliness | 10 items; score 10–40 | Severe ≤20; Moderate 21–30; Low/none 31–40. Dichotomised (loneliness vs none) for analyses | Cronbach’s α = 0.89–0.94 (original); α = 0.95 Spanish validation; test–retest r = 0.73 | Russell, 1996; Velarde-Mayol et al., 2016 |
| **TSO Social and family Assessment Scale** | Social vulnerability / social support | 5 items; score 5–25 | No social problem ≤12; Risk 13–15; Established social problem >15 | Excellent reliability ICC = 0.93 | Giménez-Bertomeu et al., 2020 |
| **Hamilton Depression Rating Scale** | Depressive symptoms severity | 17 items; score 0–52 | No depression 0–6; Mild 7–17; Moderate 18–24; Severe ≥25 | Cronbach’s α = 0.72; inter-rater reliability r = 0.99 | Hamilton, 1960; Ramos-Brieva & Cordero-Villafafila, 1988 |
| **Charlson Comorbidity Index (CCI)** | Comorbidity burden | 19 conditions; weighted score | No comorbidity 0–1; Low 2; High ≥3 | Strong predictive validity for mortality at 1 and 10 years | Charlson et al., 1987 |
| **Clinical Frailty Scale (CFS)** | Frailty / global fitness | 9-point scale | Not frail 1–3; Vulnerable 4; Frail 5–8 | Concurrent validity with FRAIL-ES r = 0.681 | Rockwood et al., 2005; Arias-Rivera et al., 2025 |
| **Barthel Index** | Functional independence (ADL) | 10 items; score 0–100 | Independent 100; Mild 91–99; Moderate 61–90; Severe ≤60 | Cronbach’s α = 0.86–0.92; inter-observer reliability κ = 0.47–1.00 | Mahoney & Barthel, 1965; Cid-Ruzafa & Damián-Moreno, 1997 |
| **Short Physical Performance Battery (SPPB)** | Physical performance/ frailty | 3 tests; score 0–12 | Not frail ≥10; Frail <10 | Cronbach’s α = 0.86 | Guralnik et al., 1994; Santamaría-Peláez et al., 2023 |
| **Downton Fall Risk Index** | Risk of falls | Score 0–11 | High risk ≥3 | Cronbach’s α = 0.57 | Downton, 1993; Aranda-Gallardo et al., 2015 |
| **EQ-5D-5L** | Health-related quality of life | 5 dimensions + EQ-VAS (0–100) | Continuous variables used in analyses | Spanish value set validated; Lin’s CCC = 0.989 | EuroQol Group, 1990; Ramos-Goñi et al., 2018 |
