# Supplementary material for: Loneliness in older women: a multidimensional population-based study of social, health, and life-course factors
Source: Front Aging. 2026 Jul 29;7:1858726. doi: 10.3389/fragi.2026.1858726 (PMC13461583; doi:10.3389/fragi.2026.1858726)
Supplement: Supplementary file 3 [file Table3.docx]

**Supplementary Table 2. Hierarchical multivariable linear regression models for loneliness (continuous UCLA Loneliness Scale score). NOT ALONE study, Spain, 2024–2025.**

| **Variable** | **Model 1 β (95% CI)** | **Model 2 β (95% CI)** | **Model 3 β (95% CI)** | **Model 4 β (95% CI)** | **Model 5 β (95% CI)** |
| --- | --- | --- | --- | --- | --- |
| **Sociodemographic domain** |  |  |  |  |  |
| *Age group (ref: 66–74 years)* |  |  |  |  |  |
| 75–84 years | -0.04 [0.10–0.38] | 0.01 [-0.5–0.09] | -0,00 [-0,07–0,06] | 0.01 [-0.05–0.08] | 0.00 [-0.06–0.07] |
| ≥85 years | 0.06 [0.04–0.17] | 0.05 [0.05–0.15] | 0.01 [-0.08–0.12] | 0.06 [-0.04–0.16] | 0.04 [-0.05–0.15] |
| *Marital status (ref: Married)* |  |  |  |  |  |
| Widowed | 0.01 [-0.08–0.11] | -0.00 [-0.10–0.09] | 0.01 [-0.08–0.10] | 0.02 [-0.06–0.11] | 0.03 [-0.05–0.11] |
| Divorced/separated/single | 0.02 [-0.08–0.13] | -0.00 [-0.10–0.10] | 0.03 [-0.06–0.13] | 0.04 [-0.04–0.14] | 0.05 [-0.04–0.14] |
| *Household composition (ref: Living with one person)* |  |  |  |  |  |
| Living alone | 0.11 [0.01–0.21] | 0.11 [0.01–0.21] | 0.10 [0.01–0.20] | 0.10 [0.02–0.19] | 0.09 [0.01–0.18] |
| Living with two or more people | 0.10 [0.00–0.19] | 0.08 [-0.00–0.17] | 0.06 [-0.01–0.15] | 0.06 [-0.01–0.14] | 0.06 [-0.01–0.14] |
| *Monthly household income (ref: €1,000–€2,500)* |  |  |  |  |  |
| <€1,000 | 0.10 [0.02–0.18] | 0.05 [-0.02–0.12] | 0.01 [-0.06–0.08] | 0.01 [-0.05–0.09] | 0.01 [-0.61–0.08] |
| >€2,500 | 0.06 [-0.05–0.17] | 0.07 [-0.04–0.19] | 0.06 [-0.04–0.18] | 0.07 [-0.03–0.17] | 0.06 [-0.04–0.16] |
| **Social domain** |  |  |  |  |  |
| *Participation in senior associations/groups (ref: Yes)* |  |  |  |  |  |
| No | — | 0.05 [-0.01–0.12] | 0.05 [-0.00–0.12] | 0.07 [0.00–0.13] | 0.06 [0.00–0.13] |
| No, but would like to participate | — | 0.11 [-0.00–0.23] | 0.07 [-0.03–0.19] | 0.08 [-0.02–0.19] | 0.05 [-0.05–0.16] |
| *Social Risk Assessment Scale (TSO) (ref: No social problem)* |  |  |  |  |  |
| Risk of social problem | — | 0.12 [-0.00–0.25] | 0.08 [-0.03–0.20] | 0.10 [-0.01–0.22] | 0.09 [-0.02–0.21] |
| Established social problem | — | 0.47 [0.23–0.70] | 0.35 [0.16–0.55] | 0.37 [0.17–0.56] | 0.32 [0.11–0.52] |
| **Clinical domain** |  |  |  |  |  |
| *Hamilton Depression Rating Scale (HDRS) (ref: No depression)* |  |  |  |  |  |
| Mild depression | — | — | 0.27 [0.18–0.36] | 0.29 [0.20–0.38] | 0.26 [0.16–0.36] |
| Moderate depression | — | — | 0.40 [0.17–0.63] | 0.45 [0.22–0.68] | 0.39 [0.14–0.63] |
| **Functional domain** |  |  |  |  |  |
| *Barthel Index (ref: Independent)* |  |  |  |  |  |
| Mild dependency | — | — | — | 0.06 -[0.04–0.16] | 0.05 [-0.04–0.16] |
| Moderate dependency | — | — | — | -0.15 [-0.23– -0.69] | -0.18 [-0.27– -0.08] |
| Severe/total dependency | — | — | — | -0.04 [-0.23–0.13] | -0.10 [-0.32–0.10] |
| **Quality-of-life domain** |  |  |  |  |  |
| *Health-related quality of life (EQ-5D), per 0.10-point increase* | — | — | — | — | -0.01 [-0.03–0.01] |
| *EQ-VAS, per 10-point increase* | — | — | — | — | 0.00 [0.00–0.00] |

Note: Linear regression with robust standard errors. Values are β coefficients with 95% confidence intervals (95% CIs). β coefficients represent the mean difference in UCLA loneliness score. Model 1: sociodemographic; Model 2: + social; Model 3: + clinical; Model 4: + functional; Model 5: + quality of life. Reference categories: age 66–74 years, married, living with one person, monthly household income €1,000–€2,500, participation in senior associations/groups, no social problem, no depression, and independent functional status where applicable.
