# Supplementary material for: Loneliness in older women: a multidimensional population-based study of social, health, and life-course factors
Source: Front Aging. 2026 Jul 29;7:1858726. doi: 10.3389/fragi.2026.1858726 (PMC13461583; doi:10.3389/fragi.2026.1858726)
Supplement: Supplementary file 4 [file Table4.docx]

**Supplementary Table S3. Final multivariable model (Model 5): adjusted prevalence ratios (aPRs), 95% confidence intervals, and individual variance inflation factors (VIFs). NOT ALONE study, Spain, 2024–2025.**

| **Variable** | **Model 5** | |
| --- | --- | --- |
|  | β (95% CI) | VIFs |
| **Sociodemographic domain** |  |  |
| *Age group (ref: 66–74 years)* |  |  |
| 75–84 years | 1.09 [0.71-1.69] | 1.33 |
| ≥85 years | 1.14 [0.64-2.03] | 1.67 |
| *Marital status (ref: Married)* |  |  |
| Widowed | 1.16 [0.70-1.93] | 2.16 |
| Divorced/separated/single | 1.30 [0.68-2.49] | 1.2 |
| *Household composition (ref: Living with one person)* |  |  |
| Living alone | 1.99* [1.13-3.50] | 2.25 |
| Living with two or more people | 1.54 [0.91-2.61] | 1.59 |
| *Monthly household income (ref: €1,000–€2,500)* |  |  |
| <€1,000 | 1.02 [0.65-1.62] | 1.23 |
| >€2,500 | 1.31 [0.62-2.80] | 1.11 |
| **Social domain** |  |  |
| *Participation in senior associations/groups (ref: Yes)* |  |  |
| No | 1.55 [0.97-2.49] | 1.36 |
| No, but would like to participate | 1.35 [0.76-2.40] | 1.36 |
| *Social Risk Assessment Scale (TSO) (ref: No social problem)* |  |  |
| Risk of social problem | 1.50 [0.93-2.41] | 1.19 |
| Established social problem | 1.59 [0.89-2.84] | 1.27 |
| **Clinical domain** |  |  |
| *Hamilton Depression Rating Scale (HDRS) (ref: No depression)* |  |  |
| Mild depression | 3.59*** [2.27-5.68] | 1.35 |
| Moderate depression | 5.37*** [2.72-10.61] | 1.23 |
| **Functional domain** |  |  |
| *Barthel Index (ref: Independent)* |  |  |
| Mild dependency | 1.39 [0.85-2.28] | 1.14 |
| Moderate dependency | 0.42**[0.24-0.74] | 1.67 |
| Severe/total dependency | 0.64 [0.27-1.54] | 1.66 |
| **Quality-of-life domain** |  |  |
| *Health-related quality of life (EQ-5D), per 0.10-point increase* | 0.96 [0.88-1.04] | 2.29 |
| *EQ-VAS, per 10-point increase* | 0.999 [0.998-1.000] | 1.45 |
| **Model fit** |  |  |
| N | 502 | |
| Log likelihood | -200.82 | |
| AIC | 451.64 | |
| BIC | 557.10 | |

Abbreviations: aPR, adjusted prevalence ratio; CI, confidence interval; VIF, variance inflation factor; HDRS, Hamilton Depression Rating Scale; TSO, Social Risk Assessment Scale; EQ-5D, EuroQol 5-Dimension; EQ-VAS, EuroQol Visual Analogue Scale. All individual VIF values were <2.5, indicating no evidence of problematic multicollinearity.
